# Medically unexplained symptoms are common in women in tertiary neurological healthcare center: A survey cohort study of persons investigated for suspected multiple sclerosis

**DOI:** 10.1002/brb3.3459

**Published:** 2024-03-07

**Authors:** Lenka Novakova, Anna Karin Hedström, Markus Axelsson, Anne Frandsen Brandt, Lars Alfredsson, Tomas Olsson, Jan Lycke

**Affiliations:** ^1^ Department of Clinical Neuroscience, Institute of Neuscience and Physiology, Sahlgrenska Academy University of Gothenburg Gothenburg Sweden; ^2^ Department of Neurology, Region Västra Götaland Sahlgrenska University Hospital Gothenburg Sweden; ^3^ Department of Clinical Neuroscience Karolinska Institute Stockholm Sweden; ^4^ Center for Molecular Medicine Karolinska University Hospital Stockholm Sweden; ^5^ Center for Occupational and Environmental Medicine, Region Stockholm Stockholm Sweden; ^6^ Institute of Environmental Medicine, Karolinska Institute Stockholm Sweden

**Keywords:** autoimmune disorder, epidemiological, functional disorder, medically unexplained symptoms, multiple sclerosis, survey

## Abstract

**Background:**

A significant proportion of individuals with suspicious onset of multiple sclerosis (MS) does not fulfill the diagnostic criteria. Although some receive other diagnoses, many remain undiagnosed and lack healthcare follow‐up. This study aimed to characterize persons with undetermined diagnosis (PwUD) through a questionnaire.

**Methods:**

Incident cases with suspected MS were consecutively admitted to a tertiary neurological healthcare center in a prospective cohort study. Those who remained undiagnosed after 40 months (mean, range 31–52) were considered PwUD. They completed a modified questionnaire, previously used in a population‐based case‐control study of incident MS cases. Their responses were compared with two control cohorts, persons with MS (PwMS) and healthy controls, randomly selected from national registries, matched by age, gender, and area of residence.

**Results:**

Out of 271 patients with suspected MS onset, 72 (20.3%) were PwUD with a female majority (79%). The response rate was 83% and 39% reported persisting MS‐like symptoms. Compared to controls (*n* = 548) and PwMS (*n* = 277), fewer PwUD were currently smoking (*p* = .4 and *p* = .03), consumed less alcohol (*p* = .04 and *p* = .01), and had children (*p* = .02 and *p* = .002). PwUD reported occurrence of other autoimmune disease in 29%, higher compared to PwMS and controls (*p* < .001 and *p* < .001).

**Conclusions:**

UD is common among persons investigated for suspected MS, in particular among female parents. Our data suggest that PwUD can be characterized as nonsmokers with low alcohol consumption and a higher prevalence of autoimmune disease, in particular thyroid disease.

## 1 INTRODUCTION

In persons with suspicious onset of multiple sclerosis (MS), a significant proportion does not fulfill the diagnostic criteria of MS (Kaisey et al., 2019; Solomon & Weinshenker, 2013; Solomon et al., 2012). Although a proportion is diagnosed with other neurological diagnoses (Novakova et al., 2018), the majority remains undiagnosed and around one third is not followed‐up in healthcare (Carmosino et al., 2005; Yamout et al., 2017). Our previous study confirmed that the risk of clinically isolated syndrome (CIS) or MS in persons not fulfilling the diagnostic criteria of MS at clinical onset is low despite persistence of neurological symptoms (Boster et al., 2008; Constantinescu et al., 2021). Conversely, revision of a neurological diagnosis as a functional disorder, is also rare (Walzl et al., 2019). However, misdiagnosis could potentially harm patients in both directions (Solomon & Klein, 2013; Solomon & Weinshenker, 2013; Solomon et al., 2012).

Medically unexplained symptoms (MUS) are usually considered to be conditioned by somatoform disorders or functional somatization. In the general population, it affects around 10% of the population and are common in the young and middle‐aged, whereas the prevalence rates seem to decline after the age of 65 years (Hilderink et al., 2013). The prevalence is higher in clinical populations, 20% to 50% in primary care and 25% to 66% in particular specialties, including neurology (Nimnuan et al., 2001). The symptoms present in different forms depending on medical specialty, are often chronic, impair everyday functioning as well as quality of life (Constantinescu et al., 2021; Nimnuan et al., 2001). Healthcare costs (Konnopka et al., 2012) are comparable to mental health problems like depression or anxiety disorders.

In our previous study, persons with undetermined diagnosis (PwUD) showed signs of impaired cognition and reduced quality of life (QoL) at symptom onset and at reassessment after mean follow‐up time of 40 months (range 31–52). Cognition and QoL remained impaired to a similar degree as in persons with MS (PwMS). We found no evidence of neurodegeneration in PwUD (Constantinescu et al., 2021). In this study, we invited PwUD to respond to an epidemiological questionnaire to further characterize this cohort.

## 2 MATERIALS AND METHODS

### 2.1 Study subjects

PwUD were identified at the Multiple Sclerosis Center at the Department of Neurology, Sahlgrenska University Hospital, Gothenburg, Sweden. Between April 2014 and June 2016, 271 individuals with clinical signs suggestive of MS onset were consecutively included, investigated at the clinic, and prospectively followed. Of these, 136 were diagnosed with CIS/MS, 46 received another diagnosis, and 89 were not diagnosed with any physical or psychiatric disease and therefore labeled PwUD (Novakova et al., 2018). The MRI was repeated after 6–12 months in PwUD if they presented with nonspecific findings. At the time of diagnostic work‐up, persons were also assessed with multiple sclerosis impact scale (MSIS‐29), grading symptoms between 1–5 (1 = not at all, 2 = a little, 3 = moderately, 4 = quite a bit, 5 = extremely), and these results have previously been published (Constantinescu et al., 2021). Forty months (range 31–52) after symptom onset, the latter group was invited to fill out a modified questionnaire used in a national population‐based case‐control study (Epidemiological Investigation of Multiple Sclerosis, EIMS), described in detail elsewhere (Alfredsson et al., 2023; Hedstrom et al., 2020; Hillert & Stawiarz, 2015). Questionnaires were obtained from 74 PwUD (response rate 83%). Of the 74 PwUD who filled out the questionnaire, another two individuals had received a medical diagnosis and these were excluded, leaving 72 PwUD in the present study.

The PwUD were compared with the following two control groups from the same geographic area: (1) 277 EIMS patients diagnosed with MS according to the McDonald criteria at the Sahlgrenska MS Center between January 2006 and June 2016 and (2) 548 EIMS controls randomly selected from the national population registry, matched to the MS cases by age in 5‐year strata, gender, and residential area. The study was approved by the Regional Ethical Review Board in Gothenburg, Sweden (895‐13); all subjects participated voluntarily and gave their informed consent. The EIMS study was approved by the Regional Ethical Review Board in Stockholm (2004/1‐4:6).

### 2.2 Questionnaire

The EIMS questionnaire includes questions regarding demographic factors, lifestyle habits, heredity for autoimmune (AI) disease and own AI disease (rheumatoid arthritis, Sjögren's syndrome, systemic lupus erythematosus, psoriasis, thyroid disorder, diabetes mellitus type 1, inflammatory bowel disorder, and vitiligo). In this modified questionnaire, used in the current study, the questions were unchanged. Only questions not relevant for persons without multiple sclerosis were omitted. We added questions about contact with other health care providers, any new diagnosis, and/or treatment and about persistence of the initial symptoms. The questions used in the present study were translated into English and are available as Supplementary Material.

### 2.3 Statistical analysis

Differences in variables between PwUD and the two control groups (MS cases and controls without MS) were assessed using one‐way analysis of variance (ANOVA) for continuous variables and the Kruskal–Wallis test (Mann–Whitney *U* test) for categorical variables.

For variables that differed between PwUD and the control groups, we calculated odds ratios (OR) with 95% confidence intervals (CI) using logistic regression models, comparing PwUD with each control group. Apart from calculating the crude OR of PwUD associated with each factor, compared to cases of MS and healthy controls, we also ran a model in which each variable was adjusted for by the other variables. Age was adjusted for in 5‐year age strata. All other variables were dichotomized. Ancestry was dichotomized into Swedish or non‐Swedish origin, parental status into having no children or being a parent, university studies into yes or no, smoking into current smoking or nonsmoking. Alcohol consumption was dichotomized using the median consumption among controls at cutoff. Autoimmune disease was dichotomized into yes or no. Level of tiredness was dichotomized into high or low based on the median value among controls. Wald Chi‐Squares test was used to analyze the proportion of patients with persisting symptoms and MRI findings. Anonymized data underlying this article will be shared on reasonable request from any qualified investigator that wants to analyze questions that are related to the published article.

## 3 RESULTS

Forty months after symptom onset, 28 (39%) of PwUD reported persisting symptoms. The initial symptoms and persisting symptoms are presented in Table 1. In the MSIS‐29, there are three questions that might indicate signs of depression: problems sleeping, feeling mentally fatigued and feeling depressed. The PwUD reported, on a scale 1–5, median of 2 (range 1–5) indicating a little problem in sleeping, median of 3 (range 1–5) indicating feeling moderately mentally fatigued and median of 2 (range 1–5) indicating a little problem with feeling depressed. No specific medical diagnoses were assigned to PwUD by any other health care provider. In a subsequent assessment in primary care, some received an explanation attributing their symptoms to either stress and/or muscular tension (*n* = 17). At the diagnostic MRI, 48 PwUD presented with nonspecific findings that were unchanged at follow‐up and did not lead to any diagnosis. The proportion of persisting symptoms were 33% in patients with normal MRI and 42% in patients with nonspecific MRI findings (*p* = .494).

**TABLE 1:** Clinical symptoms in PwUD.

	Initial symptoms	Persisting symptoms
Sensory symptoms	50	19
Multifocal symptoms^a^	7	3
Sensorimotor symptoms	5	3
Vertigo and diplopia	3	0
Motor symptoms	2	1
Vertigo	2	0
Visual impairment	2	1
Hearing loss	1	1
Total number of subjects	72	28

^a^ Combinations of sensory symptoms, vertigo, pain, and/or visual impairment.

### 3.1 Characteristics of PwUD, PwMS, and controls

The characteristics of PwUD, PwMS and controls are presented in Table 2. In the group of PwUD, there were more women [compared to MS (*p* = .04) and controls (*p* = .05)] who were parents [compared to MS (*p* = .002) and controls (*p* = .02)]. Non‐Swedish origin was more common among PwUD [compared to MS (*p* = .008) and controls (0.02)]. There were more nonsmokers among PwUD [compared to MS (*p* = .03)], and they consumed less alcohol [compared to MS (*p* = .01) and controls (*p* = .04)]. Further, they reported more tiredness [compared to controls (*p* < .001) but similar as PwMS (*p* = .2)] and they felt supported and appreciated at their work and outside home [compared to MS (*p* < .001) and controls (*p* < .001)].

**TABLE 2:** Characteristics of PwUD, PwMS, and controls.

Study	Sahlgrenska	EIMS			
Case‐control status	PwUD	PwMS	*p* Value^1^	Controls	*p* Value^2^
Total	72	277		548	
Age of onset (mean, SD) Age of onset (median, range)	34.3 (9.4) 33.0 (16–56)	33.8 (10.1) 33.0 (12–62)	.7	NA	NA
Age at study inclusion (mean, SD) Age at study inclusion (median, range)	38.5 (9.7) 37.5 (20–60)	38.3 (10.3) 36.0 (17–70)	.4	38.1 (9.9) 37.0 (17–69)	.6
Female (*n*, %)	57 (79)	185 (67)	.04	374 (68)	.05
Swedish^3^ (*n*, %)	44 (61)	209 (75)	.008*	392 (72)	.03*
Currently living with an adult (*n*, %) Lived with an adult 5 years ago (*n*, %)	52 (72) 49 (68)	206 (76) 197 (72)	.5 .5	197 (77) 391 (76)	.4 .2
Children (*n*, %) Number of children (mean, SD)	53 (74) 1.4 (1.0)	142 (52) 1.0 (1.1)	.002*	324 (59) 1.2 (1.2)	.02*
Autoimmune disease^4^ (*n*, %)	21 (29)	35 (13)	<.001*	73 (13)	<.001*
Heredity for AI disease^5^ (*n*, %)	46 (64)	195 (71)	.3	345 (63)	.8
University (*n*, %) Number of terms (mean, SD) Exam (*n*, %)	37 (51) 4.2 (5.3) 41 (57)	124 (45) 3.2 (4.3) 71 (26)	.3	249 (45) 3.3 (4.2) 149 (27)	.3
Ever smoking^6^ (*n*, %) Current smoking^9^ (*n*, %) Past smoking^10^ (*n*, %) Number of pack years (mean, SD)	29 (39) 13 (19) 16 (23) 2.3 (5.9)	1351(55) 82 (30) 69 (25) 3.5 (6.6)	.02* .03* .6 .1	240 (44) 121 (22) 119 (22) 2.7 (6.3)	.4 .4 .9 .5
Snuff use (*n*, %)	13 (18)	46 (17)	.7	95 (17)	.8
Exercise^11^ at inclusion (mean, SD) Exercise 5 years ago (mean, SD)	2.5 (1.1) 2.6 (1.1)	2.5 (0.9) 2.7 (1.0)	.6 .5	2.6 (0.9) 2.7 (1.0)	.9 .7
Low intake of fatty fish^12^ (*n*, %)	7 (10)	44 (16)	.2	93 (17)	.1
Alcohol drinkers (*n*, %) Gram alcohol/week (mean, SD)	47 (65) 29.8 (37.2)	196 (71) 45.1 (65.1)	.4 .01*	371 (68) 52.8 (97.6)	.7 .04*
Tiredness^13^ (mean, SD)	16.5 (5.1)	15.8 (4.2)	.2	14.6 (3.9)	<.001*
Trust^14^ outside home (mean, SD)	1.4 (0.6)	2.2 (1.0)	<.001*	2.2 (1.0)	<.001*
Trust at home (mean, SD)	1.5 (0.9)	1.6 (0.9)	.2	1.6 (0.8)	.2
Economy^15^ (mean, SD)	1.4 (0.7)	1.6 (0.9)	.5	1.5 (0.8)	.5

AI = autoimmune disease, EIMS = Epidemiological Investigation of Multiple Sclerosis, *n* = number, PwUD = persons with undetermined diagnosis, PwMS = persons with multiple sclerosis, SD = standard deviation.

1 = *p* value for difference between PwUD and PwMS; 2 = *p* value for difference between PwUD and controls; 3 = born in Sweden with parents who have not immigrated from outside Sweden; 4 = autoimmune disease except MS; 5 = heredity for any of the mentioned autoimmune diseases; 6 = ever smoking before index; index = year of disease onset among cases and corresponding controls, or first disease symptoms among noncases; 9 = smoking at index; 10 = past smoking at index; 1 pack year = 20 cig smoked daily during 1 year; 11 = exercise was given a value between 1 (lowest exposure) and 4 (highest exposure); 12 = fish intake never or seldom (less than monthly); 13 = each of the seven questions on tiredness was given a number ranging between 1 (disagree) and 4 (agree), an index ranging between 7 and 28 was created by adding the numbers together, questions 1, 3, 5 and 7 were reversed; 14 = feelings of trust were given a value between 1 (agree) and 4 (disagree); 15 = question on money was given a value between 1 and 4, a higher value indicates financial problems.

PwUD reported higher prevalence of autoimmune disease (*n* = 21, 29%) compared to MS cases (*n* = 35, 13%, *p* < .001) and healthy controls (*n* = 73, 13%, *p* < .001). The distribution of reported autoimmune disease is shown in Table 3. Most frequently, PwUD reported thyroid disorders (*n* = 11, 15.5%), that is, 9 females of 57 (19%) and 2 males of 15 (15%). Further, PwUD reported psoriasis *n* = 5 (6.9%), rheumatoid arthritis *n* = 4 (5.6%), vitiligo *n* = 4 (5.6%), and inflammatory bowel disease *n* = 2 (2.8%). None of PwUD reported Sjögren's syndrome, systemic lupus erythematosus and diabetes mellitus type 1.

**TABLE 3:** Reported autoimmune disease.

	PwUD, *n* (%)	PwMS, *n* (%)	Controls, *n* (%)
Rheumatoid arthritis	4 (5.6)	0	2 (0.4)
Sjögren's syndrome	0	0	0
Systemic lupus erythematosus	0	6 (2.2)	18 (3.3)
Psoriasis	5 (6.9)	15 (4.4)	27 (4.9)
Thyroidea disorder	11 (15.5)	5 (1.8)	4 (0.7)
Diabetes mellitus type 1	0	5 (1.8)	17 (3.1)
Inflammatory bowel disorder	2 (2.8)	3 (1.1)	2 (0.4)
Vitiligo	4 (5.6)	10 (3.6)	12 (2.2)

*n* = number, PwUD = persons with undetermined diagnosis, PwMS = persons with multiple sclerosis.

### 3.2 Comparing PwUD with cases of MS

The adjusted OR of UD was more than tripled among parents, compared to those who had no children (OR 3.2, 95% CI 1.6–6.5). Suffering from another autoimmune disease than MS was also associated with increased OR of UD (OR 2.9, 95% CI 1.4–5.8) as was non‐Swedish origin (OR 2.3, 95% CI 1.2–4.3) (Table 4).

**TABLE 4:** OR with 95% CI of receiving an undetermined diagnosis compared to patients diagnosed with MS.

Characteristic		PwUD/PwMS	OR (95% CI)^a^	OR (95% CI)^b^
Sex	Male	15/92	1.0 (reference)	1.0 (reference)
	Female	57 /185	**1.9 (1.0**–**3.5)**	1.6 (0.8–3.1)
Ancestry	Swedish	43/209	1.0 (reference)	1.0 (reference)
	Non‐Swedish	29/68	2.0 (1.1–3.4)	**2.3 (1.2**–**4.3**)
Parental status	No children	19/135	1.0 (reference)	1.0 (reference)
	Parent	53/142	**2.7 (1.5**–**4.7)**	**3.2 (1.6**–**6.5)**
Smoking	Nonsmoker	59/195	1.0 (reference)	1.0 (reference)
	Current	13/82	0.5 (0.3–1.0)	0.5 (0.3–1.1)
Alcohol consumption	< median	23/128	1.0 (reference)	1.0 (reference)
	≥ median	49/149	0.5 (0.3–0.9)	0.8 (0.4–1.5)
Autoimmune disease	No	51/242	1.0 (reference)	1.0 (reference)
	Yes	21/35	**2.8 (1.5**–**5.3)**	**2.9 (1.4**–**5.8)**
Level of tiredness	Low	27/115	1.0 (reference)	1.0 (reference)
	High	45/162	1.2 (0.7–2.0)	1.1 (0.1–2.9)

^a^ Crude data.

^b^ Adjusted for age in 5‐year age strata and for all other variables in the table.

PwUD = persons with undetermined diagnosis, PwMS = persons with multiple sclerosis, OR = odds ratio, CI = confidence interval.

### 3.3 Comparing PwUD with healthy controls

Being a parent and suffering from another autoimmune diseases than MS were associated with a doubled risk of receiving an undefined diagnosis. The adjusted OR of UD was 1.9 (95% CI 1.0–3.7) among parents, compared to those without children. Autoimmune disease rendered an OR of UD of 2.2 (95% CI 1.2–4.1) (Table 5).

**TABLE 5:** OR with 95% CI of receiving an undetermined diagnosis compared to healthy controls.

Characteristic		PwUD/controls	OR (95% CI)^a^	OR (95% CI)^b^
Sex	Male	15/174	1.0 (reference)	1.0 (reference)
	Female	57/374	1.8 (1.0–3.2)	1.3 (0.7–2.4)
Ancestry	Swedish	44/392	1.0 (reference)	1.0 (reference)
	Non‐Swedish	28/156	**1.6 (1.0**–**2.7)**	1.4 (0.8–2.5)
Parental status	No children	19/224	1.0 (reference)	1.0 (reference)
	Parent	53/324	**1.9 (1.1**–**3.3)**	**1.9 (1.0**–**3.7)**
Smoking	Nonsmoker	59/427	1.0 (reference)	1.0 (reference)
	Current	13/121	0.8 (0.4–1.5)	0.7 (0.4–1.4)
Alcohol consumption	< median	49/276	1.0 (reference)	1.0 (reference)
	≥ median	23/272	**0.5 (0.3**–**0.8)**	**0.5 (0.3**–**0.9)**
Autoimmune disease	No	51/475	1.0 (reference)	1.0 (reference)
	Yes	21/73	**2.7 (1.5**–**4.7)**	**2.2 (1.2**–**4.1)**
Level of tiredness	Low	27/317	1.0 (reference)	1.0 (reference)
	High	45/231	**2.5 (1.5**–**4.1)**	**2.2 (1.3**–**3.7)**

^a^ Crude data.

^b^ Adjusted for age in 5‐year age strata and for all other variables in the table.

PwUD = persons with undetermined diagnosis, OR = odds ratio, CI = confidence interval.

## 4 DISCUSSION

This study aimed to characterize the cohort of PwUD using an epidemiological survey questionnaire. The main characteristics of the studied cohort were female gender, non‐Swedish origin, well educated, nonsmokers with less alcohol consumption, having children, and reporting higher occurrence of own autoimmune disease. In previous studies, female gender, younger age and current employment were associated with higher prevalence of MUS (Nimnuan et al., 2001; Snijders et al., 2004), psychiatric diagnosis was not (Nimnuan et al., 2001; Snijders et al., 2004). However, to our knowledge, a history of autoimmune disease in patients with MUS has not been reported previously.

In our study, the high prevalence of PwUD is similar to the prevalence of MUS, around 20% (Hilderink et al., 2013; Nimnuan et al., 2001). In Dutch academic outpatient clinics for general neurology, the prevalence of MUS among newly referred patients was 35% (Snijders et al., 2004). We report high persistence of neurological symptoms (39%) during the 3‐year follow‐up. There are few follow‐up studies of MUS, the longest had a median follow‐up of 12.5 years (range 9 to 16) (Stone et al., 2003), with 83% reported remaining symptoms, often resulting in limitations in physical function and discomfort. Although, most of our patients had sensory symptoms (Constantinescu et al., 2021), which are less limiting, they may cause discomfort and increased use of health care resources (Stone et al., 2003).

Studying MUS is complicated by the heterogeneity of features and symptoms and the lack of consensus in defining the diagnosis. Furthermore, there is a risk of overdiagnosis of a disease, probably due to the risk of missing a diagnosis such as MS (Nimnuan et al., 2000). Physicians often prefer to do unnecessary investigations and tests when patients show symptoms where the working diagnosis is absent (Kiderman et al., 2013). The patients included in this study underwent a routine examination due to the suspicion of MS during the inclusion period (Novakova et al., 2018). When necessary to rule out another diagnosis, the patients were examined further, but unnecessary tests were avoided.

When diagnosing functional neurological disorder, there is recommendation to validate patients concerns with an appropriate diagnostic label (Clemente Fuentes et al., 2021). Functional somatic symptoms and disorders are complex diagnoses and challenging across medical specialties. There is a proposal for a new diagnostic classification, functional somatic disorder (FSD), as their physical symptoms have complex etiological mechanisms, neither purely somatic not purely mental (Burton et al., 2020), similar to pain diagnosis. Nowadays, patients with FSD are divided by treating specialists. However, it was shown that symptoms of FSD overlap and the differences specific for somatic syndromes are often artifacts of medical specialization (Wessely et al., 1999). PwUD included in this study did not match the typical psychosomatic patient. There was a substantial suspicion of MS after they were assessed by a neurologist at our tertiary neurology center. Therefore, our patients were not managed according to the recommendations for MUS (Husain & Chalder, 2021). However, there is a need for more effective clinical guidelines for management of persistent unexplained physical symptoms (Mayou, 1991). Complex interplay between body and mind occurs during the transition from acute to persistent somatic symptoms (Henningsen et al., 2018). A global management of patients with MUS is thus needed, including family, friends, colleagues, and caregivers to avoid the negative development that is often seen in affected individuals (Tobback et al., 2019).

The discovered characteristics of PwUD regarding socioeconomic status, social relationships, lifestyle‐related factors were favorable. Surprisingly, the higher occurrence of own autoimmune disease in this cohort has not previously been observed. However, the autoimmune disorder was not always documented in the medical records despite a thorough medical history at the initial visit. The most frequently reported autoimmune disease was primary hypothyroidism, which is a common disease affecting up to 5% of the general population (Chiovato et al., 2019), is 5 to 8 times more common in women than in men (Lauretta et al., 2018), and prevalence increases with age, with a peak incidence between the ages of 30 and 50 years (Chiovato et al., 2019). However, the increased prevalence of thyroid disorders in our cohort could not be explained by sex. The proportion of thyroid disorders among PwUD was the same in female and male, further PwUD were sex and age matched to controls.

The main limitation of our study is the selection of patients. We included only patients who sought care for suspected MS. However, patients were assessed for all potential differential diagnoses, both neurological and nonneurological. The selection of patients did not appear to affect the main results; as discussed above, the characteristics of our study cohort are in line with previous studies. All findings were based on self‐reported information, which could introduce bias if PwUD are more or less likely to report exposures compared to the control groups.

PwUD reported a higher prevalence of autoimmune disease. Previously, we investigated a broad set of proteins in cerebrospinal fluid (CSF) and plasma in PwUD using a highly sensitive proteomic immunoassay. Several protein concentrations were significantly different from those determined in PwMS but were essentially similar to those in healthy controls (Huang et al., 2020). However, we did not compare whether there were differences in CSF or plasma protein levels between patients with and without autoimmune disease. On the other hand, the frequency of concomitant autoimmune disease was the same in MS and control groups. A case‐control study using a questionnaire design indicated higher prevalence of autoimmune disease in MS patients than in the controls with a threshold significance after age adjustment (OR 1.9 (95% CI 1.0−3.5; *p* = .05) (Henderson et al., 2000). A systematic review showed increase of thyroid disease, inflammatory bowel disease and psoriasis in people with MS compared to controls (OR 1.66, *p* < .001; OR 1.56, *p* < .001; OR 1.31, *p* < .001; respectively), but no increase in rheumatoid arthritis or systemic lupus erythematosus (Dobson & Giovannoni, 2013). Thus our MS controls reported lower frequency in autoimmune disorders than expected.

Medically unexplained neurological symptoms are common (Evens et al., 2015; Nimnuan et al., 2000) and this study aimed to better characterize PwUD. Common differential diagnoses in patients investigated for suspected MS onset are migraine, fibromyalgia, nonspecific or nonlocalizing symptoms in patients with abnormal MRI, and psychogenic disorders (Solomon et al., 2016). We did not convert any PwUD to MS or to any other neurological disorder. However, a high proportion of PwUD reported that their MS‐like symptoms persisted. Most often, they reported recurrent paresthesia or persistent hypoesthesia, that is, neurological symptoms that are difficult to objectify. Although PwUD reported higher prevalence of autoimmune disease, these could not explain the initial neurological symptoms leading to inclusion in our study. The thorough clinical assessment of PwUD did not lead to an explanation or diagnosis and our survey could not identify any social or environmental trigger for their symptoms. PwUD is usually diagnosed with a functional disorder (Stone, 2009). Our study reports several features that can improve the identification of PwUD as a differential disorder in the assessment of people with suspected MS, where the lifestyle factors did not appear to increase the risk of medically unexplained symptoms. Most importantly, patients without any manifest neurological condition may have persistent neurological symptoms, sleeping problems and/or mental fatigue. They might be helped by other professional support than specialized neurology care. Our data may lead to improvement of the support and interventions for these patients in healthcare.

## AUTHOR CONTRIBUTIONS

**Lenka Novakova**: Writing—original draft; investigation; writing—review and editing; data curation. **Anna Karin Hedström**: Investigation; writing—review and editing; methodology; formal analysis. **Markus Axelsson**: Investigation; writing—review and editing. **Anne Frandsen Brandt**: Writing—review and editing; data curation; project administration. **Lars Alfredsson**: Writing—original draft; writing—review and editing; funding acquisition; methodology. **Tomas Olsson**: Funding acquisition; writing—review and editing; methodology. **Jan Lycke**: Supervision; resources; writing—review and editing; funding acquisition; conceptualization; investigation; methodology.

## CONFLICT OF INTEREST STATEMENT

AKH and AB report no conflicts of interests.

LN has received lecture honoraria from Biogen, Novartis, Teva, Sanofi and has served on advisory boards for Merck, Janssen and Sanofi. MA has received compensation for lectures and/or advisory boards from Biogen, Genzyme and Novartis. LA reports grants from Swedish Research Council, grants from Swedish Research Council for Health Working Life and Welfare, grants from Swedish Brain Foundation, during the conduct of the study; personal fees from Teva, personal fees from Biogene Idec, outside the submitted work. TO has received lecture/advisory board honoraria, and unrestricted MS research grants from Biogen, Novartis, Sanofi and Merck. JL has received travel support and/or lecture honoraria and has served on scientific advisory boards for Alexion, Almirall, Biogen, Bristol Myers Squibb, Celgene, Janssen, Merck, Novartis, Roche and Sanofi; and has received unconditional research grants from Biogen and Novartis, and financial support from Sanofi for an investigator‐initiated study.

### PEER REVIEW

The peer review history for this article is available at https://publons.com/publon/10.1002/brb3.3459.

## Supporting information

A questionnaire

## Data Availability

Anonymized data that support the findings of this study are available from the corresponding author upon reasonable request from any qualified investigator that wants to analyze questions that are related to the published article.
