# Supplementary material for: Medically unexplained symptoms are common in women in tertiary neurological healthcare center: A survey cohort study of persons investigated for suspected multiple sclerosis
Source: Brain Behav. 2024 Mar 7;14(3):e3459. doi: 10.1002/brb3.3459 (PMC10918608; doi:10.1002/brb3.3459)
Supplement: Supplementary file 1 — A questionnaire [file BRB3-14-e3459-s001.docx]

**A questionnaire**

1) Do you still have the symptoms that caused MS to be suspected?

□ Yes □ No

2) After the investigation at the MS Center, have you sought treatment for your symptoms from another healthcare provider?

□ Yes □ No

If Yes, where did you do the investigation?_____________________________________

3) Have you received any diagnosis or explanation for the symptoms? □Yes □No

If Yes, what diagnosis or explanation?_________________________________________

4) Have you received any treatment? □ Yes □ No

If Yes, which one?_________________________________________________________

5) Has the treatment had an effect? □ Yes □ No

**The questions below have been used in a survey where various factors that may be important in developing MS are investigated. By using the same questions in our survey, we want to see if there are differences between those who have MS and those who had similar symptoms but did not get the disease.**

1) Enter gender: Male □ Female □

2) Are you married/living with a partner? □ Yes □ □ No

3) 5 years ago, were you married/did you live with your partner? □ Yes □ No

4) Do you have biological children?

□ Yes, if yes, how many? Enter year of birth:

□ No

5) Were you born in Sweden?

□ Yes

□ No, if no, in which country were you born?

6) Did either of your parents immigrate to Sweden?

□ No □ Yes

If yes, from which country?

Mother (year):

Father (year):

7) Which of the following educations have you completed?

□ Elementary school

□ High school - practical

□ High school - theoretical

□ Folk high school, number of semesters: Degree:

□ University/college, number of semesters: Degree:

8) Which of the options below has suited you best?

Choose one option for each time period up to and including your current age.

If your activity varies, e.g. between summer and winter - try to estimate an average over the year.

Age A.Sedentary B.Moderate C.Moderate regular D. Higher intensity regular

15-19 □ □ □ □

20-29 □ □ □ □

30-39 □ □ □ □

40+ □ □ □ □

1. *Sedentary leisure time:*

You mostly devote yourself to reading, TV or other sedentary occupations in your spare time. You walk, cycle or otherwise move less than 2 hours a week.

1. *Moderate exercise:*

You walk, cycle or move in some other way for at least 2 hours a week, often without breaking a sweat. This includes e.g. walking or cycling to and from work, other walks, heavier housework, ordinary gardening, fishing, table tennis and bowling.

1. *Moderate, regular exercise during leisure time:*

You exercise regularly 1-2 times per week for at least 30 minutes each time with e.g. running, swimming, tennis, badminton, or any other activity that makes you sweat.

1. *Higher intensity, regular exercise and training:*

You engage in e.g. running, swimming, badminton, gymnastics or similar on average at least 3 times per week. Each session lasts at least 30 minutes each time.

**Smoking and snuff habits**

9a) Do you smoke or have you smoked?

□ No

□ Yes If yes, state all periods of regular and irregular smoking:

If you smoke regularly or have smoked regularly, put a cross in the box □

Enter time period and quantity below. Try to give an average amount. ATTENTION! Number is stated per day.

From (year) To (year) Number of cigarettes Number of cigars/ Number of

Per day Cigarillos pipe stop Per day Per day

_____ _____ _____ _____ ____

_____ _____ _____ _____ ____

_____ _____ _____ _____ ____

9b) If you smoke occasionally - e.g. at parties - or have smoked occasionally, tick the box □

Enter time period and quantity below. Try to give an average amount. ATTENTION! Number is stated per week.

From (year) To (year) Number of cigarettes Number of cigars/ Number of

Per week Cigarillos pipe stop Per week Per week

____ ____ ____ _____ ____

____ ____ ____ _____ ____

10) Do you snuff or have you snuffed?

□ No □ Yes

If yes, specify time period and amount. Try to state an average amount:

From (year) To (year) Number of doses per week

____ ______ ________________

_____ _______ ________________

**Diseases in your family**

10) Have you or someone in your family sought a doctor and been diagnosed with any of these diseases?

If you had to see a doctor for the same illness several times, state the first time.

If you are adopted, put a cross in the box □ and answer only the part about your own illness.

**Own illness**

Multiple Sclerosis (MS): □ No □ Yes, which year?

Rheumatoid arthritis (RA): □ No □ Yes, which year?

Sjogren's syndrome: □ No □ Yes, which year?

SLE (systemic lupus erythematosus): □ No □ Yes, which year?

Psoriasis: □ No □ Yes, which year?

Thyroid hormone disorders: □ No □ Yes, which year?

Juvenile diabetes (Diabetes type 1): □ No □ Yes, which year?

Ulcerative colitis or Crohn's disease: □ No □ Yes, which year?

Vitiligo (loss of skin pigment): □ No □ Yes, which year?

Other inflammatory disease: □ No □ Yes, which year?

**Family diseases:**

Multiple Sclerosis (MS): □ No □ Yes □ Don't know

If Yes, who? (e.g. father, mother, brother, sister, other relative):

Rheumatoid arthritis (RA): □ No □ Yes □ Don't know

If Yes, who? (e.g. father, mother, brother, sister, other relative):

Sjogren's syndrome: □ No □ Yes □ Don't know

If Yes, who? (e.g. father, mother, brother, sister, other relative):

SLE (systemic lupus erythematosus): □ No □ Yes □ Don't know

If Yes, who? (e.g. father, mother, brother, sister, other relative):

Psoriasis: □ No □ Yes □ Don't know

If Yes, who? (e.g. father, mother, brother, sister, other relative):

Thyroid hormone disorders: □ No □ Yes □ Don't know

If Yes, who? (e.g. father, mother, brother, sister, other relative):

Juvenile diabetes (Diabetes type 1): □ No □ Yes □ Don't know

If Yes, who? (e.g. father, mother, brother, sister, other relative):

Ulcerative colitis or Crohn's disease: □ No □ Yes □ Don't know

If Yes, who? (e.g. father, mother, brother, sister, other relative):

Vitiligo (loss of skin pigment): □ No □ Yes □ Don't know

If Yes, who? (e.g. father, mother, brother, sister, other relative):

Other inflammatory disease: □ No □ Yes □ Don't know

If Yes, who? (e.g. father, mother, brother, sister, other relative):

**Personal relationships and people around you**

11) How well does the following statement apply to you?

There is someone/some people I can turn to if I'm in trouble. People that I can easily meet, that I trust and can get real help from when I'm having a hard time.

Outside the home:

□ Completely true □ Fairly true □ Not so true □ not true at all

At home, e.g. spouse/partner/child:

□ Completely true □ Fairly true □ Not so true □ Not true at all

12) If you suddenly found yourself in an unforeseen situation where you had to raise SEK 14,000 in one week, would you be able to handle it?

□ Yes, for sure

□ Yes, probably

□ No, probably not

□ No

**Your usual way of acting and feeling**

13) How well do you think these statements fit you?

I get tired and stressed too easily:

□ Completely true □ True most times □ Not often true □ Not true

I can handle being interrupted when I'm doing work:

□ Completely true □ True most times □ Not often true □ Not true

To get something done, I have to expend more energy than most other people:

□ Completely true □ True most times □ Not often true □ Not true

I can usually concentrate even if the surroundings are disturbing:

□ Completely true □ True most times □ Not often true □ Not true

I get stressed easily when I am asked to hurry up with a work:

□ Completely true □ True most times □ Not often true □ Not true

I feel calm and confident even if I am faced with new tasks:

□ Completely true □ True most times □ Not often true □ Not true

I think that I have less energy than most other people in my circle of acquaintances:

□ Completely true □ True most times □ Not often true □ Not true

**Eating habits**

14) Which of the following best describes your diet?

Select one option per time period.

**Normal diet/mixed diet**

Eats most things, ie. all types of meat, fish, vegetables, dairy products, cereals etc.

□ Currently □ 5 years ago □ 10 years ago

**Vegetarian diet**

Eats a vegetarian diet with some animals, ie. eat eggs and dairy products (also called lacto-ovo vegetarian)

□ Currently □ 5 years ago □ 10 years ago

**Vegetarian diet + fish and shellfish**

Eats a vegetarian diet with some animals, ie. eats eggs and dairy products as well as fish and shellfish.

□ Currently □ 5 years ago □ 10 years ago

**Mediterranean diet**

Diet consisting of a lot of fruit, vegetables, legumes, lean meat, fish and shellfish, olive oil and nuts. Fatty dairy products are minimized.

□ Currently □ 5 years ago □ 10 years ago

**GI diet**

Diet with a low glycemic index, contains little or no fast carbohydrates.

□ Currently □ 5 years ago □ 10 years ago

**Other diet**  ___________________________________________________

15) How many cups of coffee have you drunk per day?

One cup = 1.5 dl (write "0" if you haven't drunk coffee).

Choose one option for each time period up to and including your current age.

Age

15-19 _____

20-29 _____

30-39 _____

40+ _____

16) How many cups of tea have you drunk per day?

One cup = 2.5 dl (write "0" if you haven't drunk tea)

Choose one option for each time period up to and including your current age.

Age Black tea Green tea Red tea

15-19 _____ _______ ______

20-29 _____ _______ ______

30-39 _____ _______ ______

40+ _____ _______ ______

17) How often, on average, have you eaten oily fish?

(e.g. herring, stream, mackerel, tuna, salmon, whitefish and char)

Choose one option for each time period up to and including your current age.

Age

15-19 years:

□ Several times/day □ Daily □ 3-6 times/week □ 1-2 times/week

□ 1-3 times/month □ less than 1 time/month

20-29 years:

□ Several times/day □ Daily □ 3-6 times/week □ 1-2 times/week

□ 1-3 times/month □ less than 1 time/month

30-39 years:

□ Several times/day □ Daily □ 3-6 times/week □ 1-2 times/week

□ 1-3 times/month □ less than 1 time/month

40+

□ Several times/day □ Daily □ 3-6 times/week □ 1-2 times/week

□ 1-3 times/month □ less than 1 time/month

18) Have you taken vitamins?

Choose one option for each time period up to and including your current age.

Regularly = 3-7 tablets/week.

Occasionally = 1-2 tablets/week or less than 100 tablets/year.

Never = none or seldom

Age:

15-19 □ Yes, regularly □ Yes, occasionally □ Never

20-29 □ Yes, regularly □ Yes, occasionally □ Never

30-39 □ Yes, regularly □ Yes, occasionally □ Never

40+ □ Yes, regularly □ Yes, occasionally □ Never

19) Have you taken vitamin D supplements?

ATTENTION! Supplements that only contain vitamin D or vitamin D and calcium in combination.

Choose one option for each time period up to and including your current age.

Age:

15-19 □ Yes, regularly □ Yes, occasionally □ Never

20-29 □ Yes, regularly □ Yes, occasionally □ Never

30-39 □ Yes, regularly □ Yes, occasionally □ Never

40+ □ Yes, regularly □ Yes, occasionally □ Never

**Alcohol habits**

20) How much alcohol have you drunk on average during a typical week?

Write "0" on the types of alcohol that you do not drink/have drunk.

If you have not drunk alcohol at all during a period, put a cross in the respective box.

Number of glasses/week

Age: 15-19 years 20-29 years 30-39 years 40+ years

No alcohol □ □ □ □

Medium-strong beer □ □ □ □

Export beer/strong beer/cider

□ □ □ □

Red wine □ □ □ □

White wine □ □ □ □

Fortified wine □ □ □ □

Spirits □ □ □ □

**Profession and employment**

21) Which of the following options is true for you?

Multiple options can be specified.

Full time Part time % From year

□ Student □ □ ____ ________

□ Working as an employee □ □ ____ ________

□ Self-employed □ □ ­­­­____ _______

□ Sick leave □ □ ____ _______

□ Off duty for more than 3 months (also for studies and parental leave)

□ Unemployed for more than 3 months

□ Has activity allowance/sickness allowance (former disability pension)

□ Takes care of the household

□ Other:

22) If you study: What do you study?

Enter your field of study as accurately as possible. If you study several subjects, state this.

Instead of science, write e.g. geology, biochemistry, theoretical physics.

Instead of language, write e.g. French, interpreter training in Spanish, Swedish as a foreign language.

□ University/College □ High school □ Other: _____________

Study description: _________________________________________________

City: ____________________________________________________________

23) If you are employed or have your own business: What is your profession/job duties?

Try to be specific by leaving a professional title that describes your role in the workplace.

Even in which industry you work.

Instead of assistant write e.g. purchasing assistant, accounting assistant, advertising assistant.

Instead of factory workers write e.g. car fitter, builder of electronic devices, packer.

Instead of teacher write e.g. preschool teacher, primary school teacher, textile teacher.

Professional title: __________________________________________________

Industry: _________________________________________________________

City: ____________________________________________________________

24) What main occupations have you had during your adult life?

(From 15 years and up)

Our analysis is based solely on the information you provide. Only include occupations or employment that you have had for more than a year. We have left room for 11 different periods - when you e.g. changed workplace, duties, studied or been unemployed, you start the next period.

If you have not had any employment or employment that lasted more than one year, put a cross in the box □

If you have not worked for any period, tick the box "Other" and state what you did, e.g. parental leave, unemployed, student, off duty, etc.

Period 1:

From (year) – Up to (year)

□ Employee □ Full-time □ Part-time _____ %

□ Entrepreneur/Self-employed □ Full-time □ Part-time _____ %

□ Other:_________________ □ Full-time □ Part-time _____ %

Professional title:_______________________________________________

Industry:______________________________________________________

City:_________________________________________________________

Period 2:

From (year) – Up to (year)

□ Employee □ Full-time □ Part-time _____ %

□ Entrepreneur/Self-employed □ Full-time □ Part-time _____ %

□ Other:_________________ □ Full-time □ Part-time _____ %

Professional title:_______________________________________________

Industry:______________________________________________________

City:_________________________________________________________

Period 3:

From (year) – Up to (year)

□ Employee □ Full-time □ Part-time _____ %

□ Entrepreneur/Self-employed □ Full-time □ Part-time _____ %

□ Other:_________________ □ Full-time □ Part-time _____ %

Professional title:_______________________________________________

Industry:______________________________________________________

City:_________________________________________________________

Period 4:

From (year) – Up to (year)

□ Employee □ Full-time □ Part-time _____ %

□ Entrepreneur/Self-employed □ Full-time □ Part-time _____ %

□ Other:_________________ □ Full-time □ Part-time _____ %

Professional title:_______________________________________________

Industry:______________________________________________________

City:_________________________________________________________

Period 5:

From (year) – Up to (year)

□ Employee □ Full-time □ Part-time _____ %

□ Entrepreneur/Self-employed □ Full-time □ Part-time _____ %

□ Other:_________________ □ Full-time □ Part-time _____ %

Professional title:_______________________________________________

Industry:______________________________________________________

City:_________________________________________________________

Period 6:

From (year) – Up to (year)

□ Employee □ Full-time □ Part-time _____ %

□ Entrepreneur/Self-employed □ Full-time □ Part-time _____ %

□ Other:_________________ □ Full-time □ Part-time _____ %

Professional title:_______________________________________________

Industry:______________________________________________________

City:_________________________________________________________

Period 7:

From (year) – Up to (year)

□ Employee □ Full-time □ Part-time _____ %

□ Entrepreneur/Self-employed □ Full-time □ Part-time _____ %

□ Other:_________________ □ Full-time □ Part-time _____ %

Professional title:_______________________________________________

Industry:______________________________________________________

City:_________________________________________________________

Period 8:

From (year) – Up to (year)

□ Employee □ Full-time □ Part-time _____ %

□ Entrepreneur/Self-employed □ Full-time □ Part-time _____ %

□ Other:_________________ □ Full-time □ Part-time _____ %

Professional title:_______________________________________________

Industry:______________________________________________________

City:_________________________________________________________

Period 9:

From (year) – Up to (year)

□ Employee □ Full-time □ Part-time _____ %

□ Entrepreneur/Self-employed □ Full-time □ Part-time _____ %

□ Other:_________________ □ Full-time □ Part-time _____ %

Professional title:_______________________________________________

Industry:______________________________________________________

City:_________________________________________________________

Period 10:

From (year) – Up to (year)

□ Employee □ Full-time □ Part-time _____ %

□ Entrepreneur/Self-employed □ Full-time □ Part-time _____ %

□ Other:_________________ □ Full-time □ Part-time _____ %

Professional title:_______________________________________________

Industry:______________________________________________________

City:_________________________________________________________

Period 11:

From (year) – Up to (year)

□ Employee □ Full-time □ Part-time _____ %

□ Entrepreneur/Self-employed □ Full-time □ Part-time _____ %

□ Other:_________________ □ Full-time □ Part-time _____ %

Professional title:_______________________________________________

Industry:______________________________________________________

City:_________________________________________________________

**Working conditions**

25) Are you currently employed and/or were you employed 5 or 10 years ago?

Today: □ No □ Yes If no, skip the following questions.

5 years ago: □ No □ Yes

10 years ago: □ No □ Yes

26) Have you worked overtime?

Never: □ Today □ 5 years ago □ 10 years ago

Occasionally: □ Today □ 5 years ago □ 10 years ago

Often: □ Today □ 5 years ago □ 10 years ago

Enter average number of overtime hours/week:

□ Today____ □ 5 years ago_____ □ 10 years ago______

27) How are the requirements for professional knowledge in your work consistent with the knowledge you yourself have?

The requirement have been much higher than my own professional knowledge:

□ Today □ 5 years ago □ 10 years ago

The requirements have been somewhat higher than my own professional knowledge:

□ Today □ 5 years ago □ 10 years ago

The requirements have matched my own professional knowledge:

□ Today □ 5 years ago □ 10 years ago

The requirements have been much lower than my own professional knowledge:

□ Today □ 5 years ago □ 10 years ago

28) Have you had the opportunity to learn something new and develop at work?

Yes, to a great extent

□ Today □ 5 years ago □ 10 years ago

Yes, to some extent

□ Today □ 5 years ago □ 10 years ago

Yes, to a small extent

□ Today □ 5 years ago □ 10 years ago

No not at all

□ Today □ 5 years ago □ 10 years ago

**Physical work load**

29) How physically demanding have you usually found your work?

Choose one option for each time period.

Very, very easy:

□ Today □ 5 years ago □ 10 years ago

Very easy:

□ Today □ 5 years ago □ 10 years ago

Quite easy:

□ Today □ 5 years ago □ 10 years ago

Quite hard:

□ Today □ 5 years ago □ 10 years ago

Hard:

□ Today □ 5 years ago □ 10 years ago

Very hard:

□ Today □ 5 years ago □ 10 years ago

Very, very hard:

□ Today □ 5 years ago □ 10 years ago

30) How much of the working day have you been sedentary?

Choose an option for each time period.

Not at all:

□ Today □ 5 years ago □ 10 years ago

¼ of the time:

□ Today □ 5 years ago □ 10 years ago

¾ of the time:

□ Today □ 5 years ago □ 10 years ago

Continuously:

□ Today □ 5 years ago □ 10 years ago

**Organization and content of the work**

31) Have you been involved in decisions regarding the planning of your work?

Always:

□ Today □ 5 years ago □ 10 years ago

Usually:

□ Today □ 5 years ago □ 10 years ago

Usually not:

□ Today □ 5 years ago □ 10 years ago

Never:

□ Today □ 5 years ago □ 10 years ago

32) Do you think your work is meaningful?

Yes always.

□ Today □ 5 years ago □ 10 years ago

Yes, mostly.

□ Today □ 5 years ago □ 10 years ago

Yes, to some extent.

□ Today □ 5 years ago □ 10 years ago

No, not at all.

□ Today □ 5 years ago □ 10 years ago

33) Do you consider that your work has been positively evaluated by others?

Yes always.

□ Today □ 5 years ago □ 10 years ago

Yes, mostly.

□ Today □ 5 years ago □ 10 years ago

Yes, to some extent.

□ Today □ 5 years ago □ 10 years ago

No, not at all.

□ Today □ 5 years ago □ 10 years ago

34) Have you received help from your superiors when you have difficulties in your work?

Yes always.

□ Today □ 5 years ago □ 10 years ago

Yes, mostly.

□ Today □ 5 years ago □ 10 years ago

Yes, to some extent.

□ Today □ 5 years ago □ 10 years ago

No, not at all.

□ Today □ 5 years ago □ 10 years ago

35) Has your work required you to work very fast?

Yes, often.

□ Today □ 5 years ago □ 10 years ago

Yes, sometimes.

□ Today □ 5 years ago □ 10 years ago

No, rarely.

□ Today □ 5 years ago □ 10 years ago

No, almost never.

□ Today □ 5 years ago □ 10 years ago

36) Has your job required you to work very hard?

Yes, often.

□ Today □ 5 years ago □ 10 years ago

Yes, sometimes.

□ Today □ 5 years ago □ 10 years ago

No, rarely.

□ Today □ 5 years ago □ 10 years ago

No, almost never.

□ Today □ 5 years ago □ 10 years ago

37) Has your work required too much effort?

Yes, often.

□ Today □ 5 years ago □ 10 years ago

Yes, sometimes.

□ Today □ 5 years ago □ 10 years ago

No, rarely.

□ Today □ 5 years ago □ 10 years ago

No, almost never.

□ Today □ 5 years ago □ 10 years ago

38) Have you had enough time to complete the tasks?

Yes, often.

□ Today □ 5 years ago □ 10 years ago

Yes, sometimes.

□ Today □ 5 years ago □ 10 years ago

No, rarely.

□ Today □ 5 years ago □ 10 years ago

No, almost never.

□ Today □ 5 years ago □ 10 years ago

39) Have there often been conflicting demands in your work?

Yes, often.

□ Today □ 5 years ago □ 10 years ago

Yes, sometimes.

□ Today □ 5 years ago □ 10 years ago

No, rarely.

□ Today □ 5 years ago □ 10 years ago

No, almost never.

□ Today □ 5 years ago □ 10 years ago

40) Have you learned new things in your work?

Yes, often.

□ Today □ 5 years ago □ 10 years ago

Yes, sometimes.

□ Today □ 5 years ago □ 10 years ago

No, rarely.

□ Today □ 5 years ago □ 10 years ago

No, almost never.

□ Today □ 5 years ago □ 10 years ago

41) Is skill required in your job?

Yes, often.

□ Today □ 5 years ago □ 10 years ago

Yes, sometimes.

□ Today □ 5 years ago □ 10 years ago

No, rarely.

□ Today □ 5 years ago □ 10 years ago

No, almost never.

□ Today □ 5 years ago □ 10 years ago

42) Is creativity required in your job?

Yes, often.

□ Today □ 5 years ago □ 10 years ago

Yes, sometimes.

□ Today □ 5 years ago □ 10 years ago

No, rarely.

□ Today □ 5 years ago □ 10 years ago

No, almost never.

□ Today □ 5 years ago □ 10 years ago

43) Has your work involved doing repetitive tasks?

Yes, often.

□ Today □ 5 years ago □ 10 years ago

Yes, sometimes.

□ Today □ 5 years ago □ 10 years ago

No, rarely.

□ Today □ 5 years ago □ 10 years ago

No, almost never.

□ Today □ 5 years ago □ 10 years ago

44) Have you had the freedom to decide how to perform your work assignments

Yes, often.

□ Today □ 5 years ago □ 10 years ago

Yes, sometimes.

□ Today □ 5 years ago □ 10 years ago

No, rarely.

□ Today □ 5 years ago □ 10 years ago

No, almost never.

□ Today □ 5 years ago □ 10 years ago

45) Have you had the freedom to decide what should be performed in your job?

Yes, often.

□ Today □ 5 years ago □ 10 years ago

Yes, sometimes.

□ Today □ 5 years ago □ 10 years ago

No, rarely.

□ Today □ 5 years ago □ 10 years ago

No, almost never.

□ Today □ 5 years ago □ 10 years ago
